# Supplementary material for: Health Technology Reassessment: Addressing Uncertainty in Economic Evaluations of Oncology Drugs at Time of Reimbursement Using Long-Term Clinical Trial Data
Source: Curr Oncol. 2023 Jul 10;30(7):6596–608. doi: 10.3390/curroncol30070484 (PMC10378704; doi:10.3390/curroncol30070484)
Supplement: Supplementary file 1 [file curroncol-30-00484-s001.zip › curroncol-2477412-supplementary.pdf]

Supplementary Materials

Table S1. Model input parameter values

Input parameter	Base case value		Source
Modeling approach			
Discount Factor (costs)	3%		I.C.E.R. guidelines
Discount Factor (outcomes)	3%		
Starting age of cohort (years)	63		Robert 2015
Average body weight (kg), pembrolizumab	112.0		Wang 2017
Average body weight (kg), ipilimumab	98.7		Wang 2017
Time Horizon (months)	240		Wang 2017
Fitted parametric curve selections			
Pembrolizumab PFS	Log-logistic		Calculated
Pembrolizumab OS	Log-normal		Calculated
Ipilimumab PFS	Loglogistic		Calculated
Ipilimumab OS	Log-normal		Calculated
Unit costs			
Pembrolizumab (per mg)	$55.42		CMS 2023
Ipilimumab (per mg)	$165.45		CMS 2023
Chair time unit cost	$144.39		CMS 2023
Pre-progression patient management (monthly)	$873.37		Tarhini 2015, CPI-adjusted
Post-progression patient management (monthly)	$2,722.63		Tarhini 2015, CPI-adjusted
End of life costs	$6,484.45		Tarhini 2015, CPI-adjusted
Adverse event management costs (grade 3+)			
Fatigue	$4,350.38		CMS 2023
Diarrhea	$7,159.38		CMS 2023
Rash	$4,350.38		CMS 2023
Pruritus	$4,350.38		CMS 2023
Nausea	$7,159.38		CMS 2023
Asthenia	$0.00		Assumption
Arthralgia	$4,350.38		CMS 2023
Vitiligo	$0.00		Assumption
Colitis	$7,159.38		CMS 2023
Hepatitis	$4,350.38		CMS 2023
Hypophysitis	$7,159.38		CMS 2023
Pneumonitis	$4,350.38		CMS 2023
Adverse event rates	Pembrolizumab arm	Ipilimumab arm	
Fatigue	0.4%	1.2%	Robert 2015

Diarrhea	1.1%	3.1%	Robert 2015
Rash	0.0%	0.8%	Robert 2015
Pruritus	0.0%	0.4%	Robert 2015
Nausea	0.0%	0.8%	Robert 2015
Asthenia	0.4%	0.4%	Robert 2015
Arthralgia	0.4%	0.8%	Robert 2015
Vitiligo	0.0%	0.0%	Robert 2015
Colitis	2.5%	7.0%	Robert 2015
Hepatitis	1.8%	0.4%	Robert 2015
Hypophysitis	0.4%	1.6%	Robert 2015
Pneumonitis	0.4%	0.4%	Robert 2015
Utility values			
Progression-free health state	0.83		Wang 2017
Post-progression health state	0.78		Wang 2017

Abbreviations: CMS, Centers for Medicare and Medicaid Services; CPI, consumer price index; I.C.E.R., Institute for Clinical and Economic Review; kg, kilogram; mg, milligram

Table S2. Scenario analyses

A – Scenario analyses based on interim data from KEYNOTE-006

**Scenario analysis (based on interim data)**	**ICUR ($/QALY)**	**% change from base case**
BASE CASE (deterministic)	*$111,861*	-
Best-fitting parametric functions from Wang et al	$109,715	-2%
Parametric distributions from NICE (all Gompertz)	*survival curves cross*	
Average patient weight set equal	$101,914	-10%
Proportion of progression-free pembrolizumab patients receiving re-challenge (CADTH: 25%)	$107,792	-4%
Proportion of progression-free pembrolizumab patients receiving re-challenge (Robert 2019: 19%)	$106,731	-5%
Pembrolizumab as treat-to-progression	$175,300	36%

Abbreviations: CADTH, Canadian Agency for Drugs and Technologies in Health; ICUR, incremental cost-utility ratio; NICE, National Institute for Health and Care Excellence; QALY, quality-adjusted life-year

B – Scenario analyses based on long-term follow-up data from KEYNOTE-006

**Scenario analysis (based on long-term data)**	**ICUR ($/QALY)**	**Percent change from base case**
BASE CASE (deterministic)	*$156,829*	-
Best-fitting parametric functions from Wang et al	*survival curves cross*	
Parametric distributions from NICE (all Gompertz)	$157,932	1%
Average patient weight set equal	$147,272	-6%
Proportion of progression-free pembrolizumab patients receiving re-challenge (CADTH: 25%)	$146,604	-7%
Proportion of progression-free pembrolizumab patients receiving re-challenge (Robert 2019: 19%)	$143,936	-8%
Pembrolizumab as treat-to-progression	$425,298	171%

Abbreviations: CADTH, Canadian Agency for Drugs and Technologies in Health; ICUR, incremental cost-utility ratio; NICE, National Institute for Health and Care Excellence; QALY, quality-adjusted life-year

Figure S1. Deterministic sensitivity analyses

A – One-way sensitivity analyses based on interim data from KEYNOTE-006

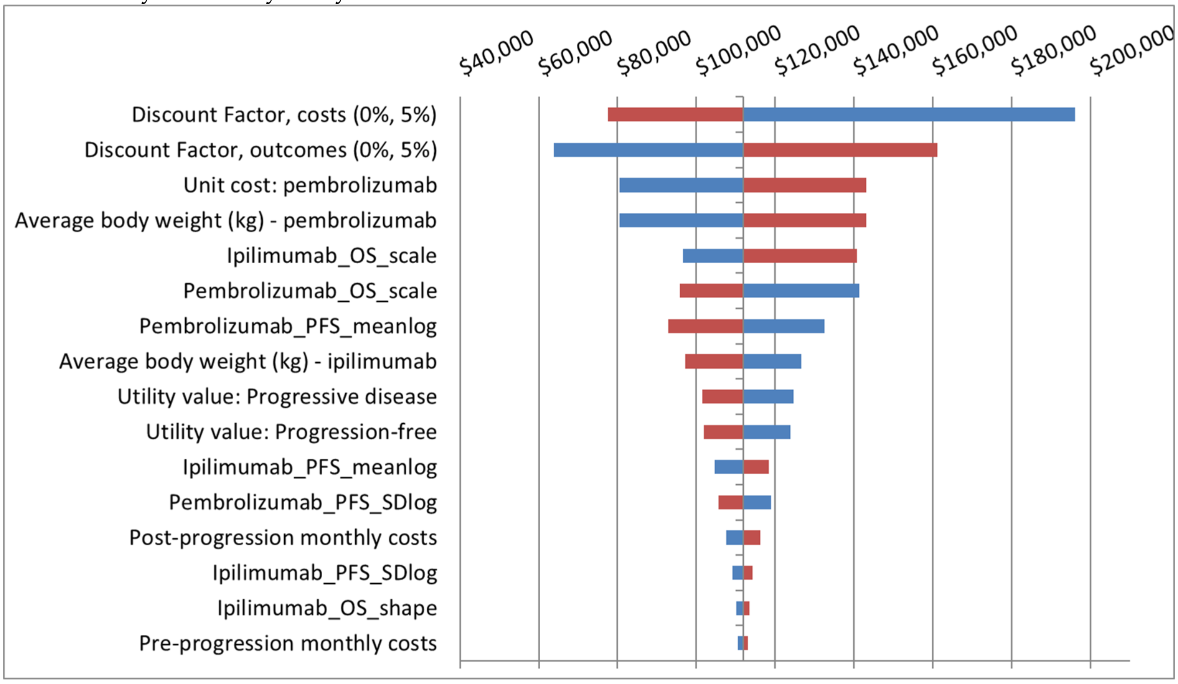

Abbreviations: kg, kilogram; OS, overall survival; PFS, progression-free survival

B – One-way sensitivity analyses based on long-term follow-up data from KEYNOTE-006

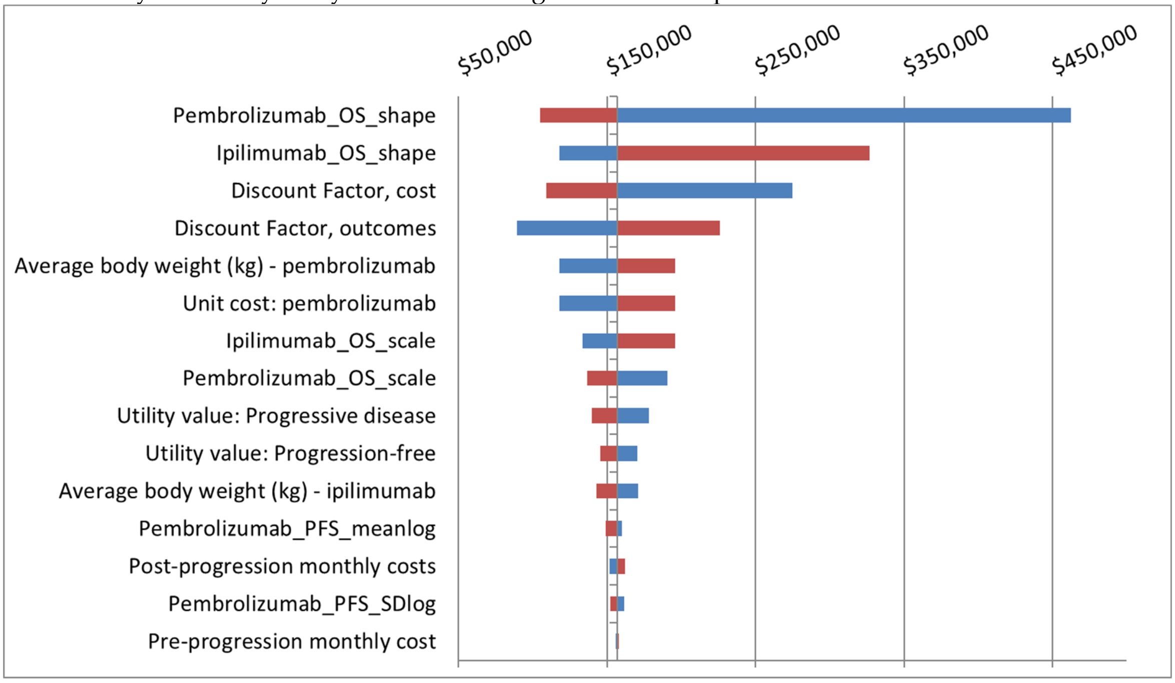

Abbreviations: kg, kilogram; OS, overall survival; PFS, progression-free survival

Figure S2. Overall survival based on long-term follow-up data from KEYNOTE-006 and pooled long-term ipilimumab data from Schadendorf et al. 2015

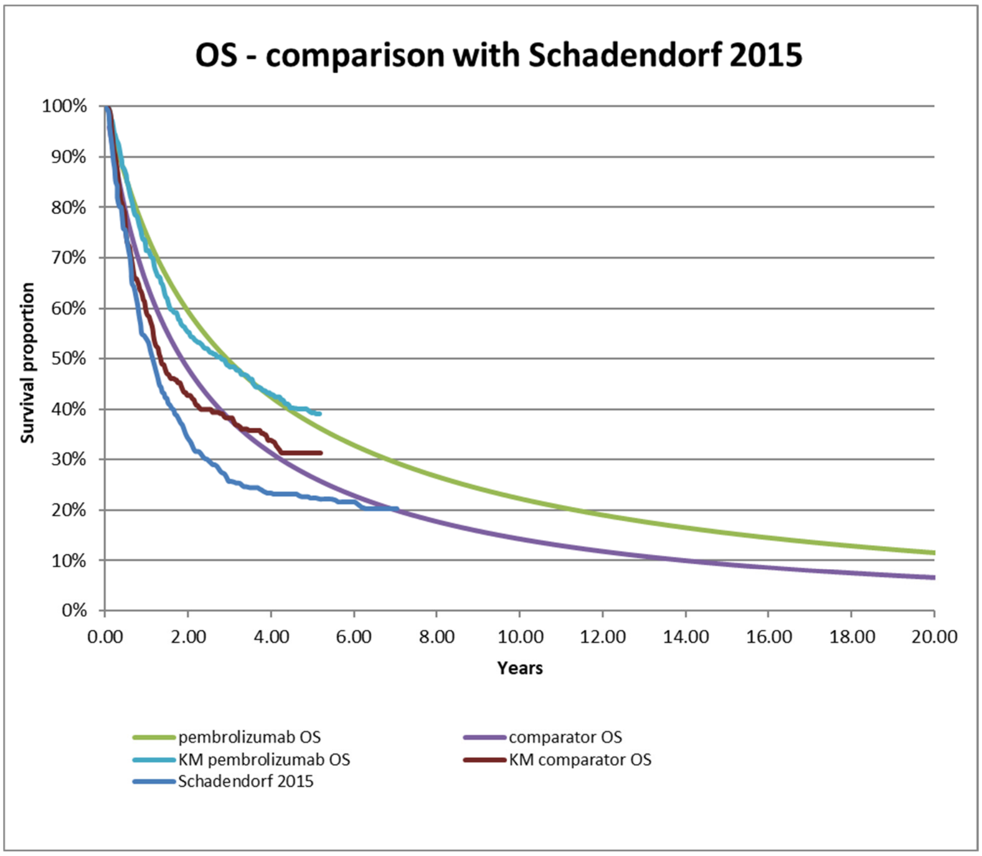

Abbreviations: KM, Kaplan-Meier; OS, overall survival; PFS, progression-free survival
